# Are Members of the Fungal Genus *Pneumocystis* (a) Commensals; (b) Opportunists; (c) Pathogens; or (d) All of the Above?

**DOI:** 10.1371/journal.ppat.1001009

**Published:** 2010-09-23

**Authors:** Melanie T. Cushion

**Affiliations:** 1 University of Cincinnati College of Medicine, Cincinnati, Ohio, United States of America; 2 The Cincinnati Veterans Affairs Medical Center, Cincinnati, Ohio, United States of America; University of California San Francisco, United States of America

## What Kind of Fungi Are *Pneumocystis*?

Members of the genus *Pneumocystis* are microscopic yeast-like fungi that reside in the lungs of almost every mammal that has been evaluated for their presence. They grow extracellularly in the alveoli of mammals and are considered to be host obligate, as they cannot grow outside the lung on artificial media. *Pneumocystis* species (spp.) are typically restricted to the lungs, although extra-pulmonary manifestations have been reported [1]. Considered to be zoonotic (able to infect more than one mammalian species), these organisms once were referred to as a single genus and species, “*Pneumocystis carinii*”. It is now clear that distinct species of *Pneumocystis* infect different mammalian hosts. Each mammalian species has at least one species of *Pneumocystis* that it harbors which cannot infect another mammalian species (species specificity).

*Pneumocystis* spp. have suffered many identity crises, beginning with their initial identification in 1909, when they were thought to be part of the life cycle of the protozoan parasite *Trypanosoma cruzi* [2]. In 1914, they were provided an identity of their own and given the genus and species designation “*Pneumocystis carinii*”, which reflected their preference for the lung, “*pneumo*”-; their characteristic morphological form, the cyst, -“*cystis*”; and “*carinii*” to honor the Italian investigator, Antonio Carini, who provided the slides for study. Presumed to be a protozoan parasite, the potential fungal nature of *P. carinii* was first raised in the 1950s, and the controversy over their protozoan or fungal nature continued into the late 20th century [1], [2]. Phylogenetic analyses first based on the nuclear small subunit rRNA sequence alignments then with additional gene sequence comparisons showed that the closest extant relatives to *P. carinii* were the fission yeast *Schizosaccharomyces pombe* and the plant pathogen *Taphrina deformans* [3].

Molecular biological techniques have been essential for providing the breadth of knowledge that is currently available for these intractable fungi. Phylogenetic determinations based on gene sequences not only permitted the fungal identity of the genus to be clarified, but provided the basis for species distinctions within the genus. Although it is anticipated that there are thousands of *Pneumocystis* species, only five have been formally described: *P. carinii* and *Pneumocystis wakefieldiae*, which infect rats; *Pneumocystis murina* in mice; *Pneumocystis oryctolagi* in rabbits; and *Pneumocystis jirovecii*, which infects humans [2].

It should be noted that the nomenclature of *Pneumocystis* has not been without controversy, particularly with the name *P. jirovecii* [2]. A number of clinician investigators raised concerns about the change from *P. carinii* to *P. jirovecii*, which had the potential to cause confusion among patients and clinicians alike [4]. However, validation of the names through the formal guidelines recommended by the fungal community combined with scientific and biological evidence and recommendations by authorities have contributed to a general acceptance of the current nomenclature, including *P. jirovecii* [5].

## Where Are They Found and How Do They Replicate?

*Pneumocystis* spp. are considered ubiquitous fungi and are found in the lungs of terrestrial mammals from almost all geographic regions with the possible exception of the Arctic and Antarctica, where their presence has not yet been surveyed. Definitive studies of *Pneumocystis* in marine mammals have not been reported. No environmental forms of *Pneumocystis* have been identified providing additional evidence that they are dependent upon their hosts for growth and transmission without need of an intermediate vector or requirement for maturation outside mammals. Human beings develop antibodies to *P. jirovecii* by the age of 4 years, but likely are exposed at a much earlier age [1]. The first exposure during the neonatal period seems to result in mild or no clinical symptoms and is probably mistaken for another infectious agent [6]. Experiments using newborn rats showed that DNA from *P. carinii* was present in their oral cavities 1–2 hours after birth, even before the first feeding, providing evidence that mammals contact *Pneumocystis* early in life [7]. This first contact may be by an airborne route where an infectious propagule is inhaled by the neonate or by intimate contact such as grooming.

**Figure 1:**
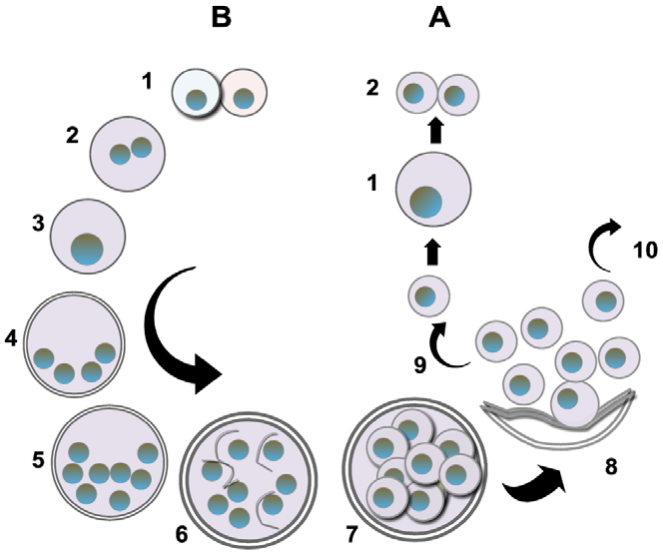
Putative life cycle *of Pneumocystis*. The entry of *Pneumocystis* into the mammalian lung likely occurs during the first year after birth. The agent of infection is suspected to be airborne spores. Recent studies suggest that the cyst/ascus (containing eight spores) may be the agent of infection [9]. After inhalation, the spores ultimately take residence in the terminal portion of the respiratory tree, the alveoli. Neither the mechanism of migration to the alveoli nor the form in which the organism arrives in the alveoli (intact ascus or individual spores) is known. (A) Asexual phase: Haploid trophic forms are thought to replicate asexually by binary fission, whereby the nuclear content is duplicated (1) along with cellular contents that (2) divide into two haploid trophic forms. (B) Sexual phase: Two presumptive mating types conjugate (1), undergo karyogamy (2), and produce a diploid zygote (3) that progresses through meiosis to produce four haploid nuclei (4) followed by an additional mitosis to produce eight nuclei (5). The nuclei are packaged into spores by invagination of the ascus cell membranes (6) to produce eight double-membrane spores (7). After completion, excystment occurs via a protunicate release by unknown mechanisms (8). The released spores become the vegetative forms that can then undergo asexual (9) or sexual replication with a presumed opposite mating type (10). The mechanism of exit out of the lung and the life cycle form that transits into the environment are unknown (the life cycle was composed using SmartDraw 10, San Diego, California).

**Figure 2:**
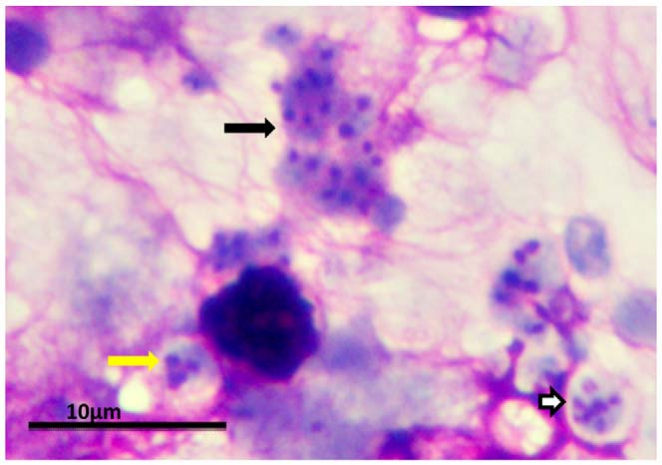
*P. murina* stained with rapid Wright-Giemsa. Clusters of *P. murina* from a homogenate of an infected mouse lung were dropped on glass slides and stained with a rapid Wright-Giemsa. The black arrow points to a cluster of trophic forms. The white arrow indicates a mature cyst. The yellow arrow indicates an immature cyst with only three nuclei present in this section. The magnification bar represents 10 um. The micrograph was taken with an Olympus BH2 microscope and DP-72 digital camera.

The life cycle of *Pneumocystis* has not been completely defined, but most putative schemes contain an asexual mode of replication via binary fission of the trophic form and a sexual mode resulting in formation of an ascus (cyst) containing eight ascospores (Figure 1). Unlike most other yeast, *Pneumocystis* spp. do not undergo budding. Mating is likely mediated by the trophic forms, as evidenced by the expression of a pheromone receptor protein on the surface of some trophs [8]. Besides the cyst and trophic forms, there are several intermediate stages that likely represent the progression from zygote through meiosis with an additional mitotic step to produce eight nuclei, followed by separation into ascospores (Figures 1 and 2). Members of the *Pneumocystis* genus are distinct from other medically significant fungi because they appear to undergo sexual replication in their mammalian hosts, who are then able to transmit the infection, unlike other fungi (e.g., *Aspergillus*) where the host is a dead end.

After inhalation, the infection is thought to be initiated by attachment of the trophic forms to the Type I pneumocyte in the host alveoli. Investigators in the field have long pondered the identity of the infectious propagule without any clear answers. However, recent studies provide evidence that the cyst may be the agent of transmission. Treatment of *Pneumocystis-*infected mice and rats with echinocandins (drugs that inhibit β-1,3-D-glucan synthesis) were shown to eliminate cysts, which contain glucan in their walls [9]. The cyst-depleted animals that contained large numbers of viable trophic forms were unable to transmit the infection by the airborne route to uninfected animals, while their untreated counterparts containing cysts were able to do so, implicating the cyst as the transmissive particle. The cyst would be an efficient way to initiate infection, as a single cyst contains eight spores. Once in the alveoli, clusters of organisms grow out into the lumen from trophic forms anchored to the Type I cells. The infection spreads throughout the entire lungs, but the manner in which the organisms move from alveolus to alveolus is not known. Recent evidence describing the ability of *P. carinii* and *P. murina* to form biofilms suggests this mechanism may be one way in which the infection can spread [10].

## Are These Fungi Pathogenic and Do They Cause Disease?

*Pneumocystis* spp. cause pneumonia in immunologically impaired mammals, which is oftentimes lethal if untreated. *P. jirovecii* pneumonia (PCP) was a leading cause of morbidity and mortality in patients with AIDS during the last two decades [11]. With the introduction of highly active anti-retroviral therapy (HAART) in 1996, the incidence of PCP has decreased in industrialized countries like the United States and England, but PCP remains the leading opportunistic infection among patients with HIV and a serious clinical problem [12]. Strikingly, the mortality rate associated with PCP before and after initiation of HAART has not changed significantly in the US from an average of about 10% [12]. Mortality is even higher in developing countries and within urban areas of the US, despite the availability of HAART [13]. In HIV-positive and other immunosuppressed patients with PCP requiring medical intensive care in Thailand, the mortality rate was about 64%, while in an inner city population in Atlanta, Georgia, the mortality rate in 1996–2006 was 37%. In patients requiring aggressive intervention such as mechanical ventilation, the rate was over 80%. PCP occurs in patients with other immunosuppressive diseases and in those whose immune systems have been debilitated by drugs such as long-term administration of corticosteroids. In fact, there has been little improvement in mortality in patients with cancer and other non-HIV diseases, and in many cases these patients fared worse than those with HIV. In one study, the mortality in non-HIV patients from PCP was 48% versus 17% in individuals with HIV [14]. The recent detection of *P. jirovecii* in new patient populations suggests that *P. jirovecii* is taking advantage of these evolving niches in the human population. Anti-tumor necrosis factor-alpha (anti-TNFα) therapy, such as inflixamab, is now commonly used to treat rheumatoid arthritis and Crohn’s disease. A recent survey of the US Food and Drug Administration Adverse Event Reporting System for *P. jirovecii* pneumonia from 1998 through 2003 identified 84 cases of PCP associated with inflixamab therapy, 27% of which resulted in death [15].

Standard antifungal drugs targeting ergosterol and ergosterol biosynthesis, such as amphotericin B and the azoles, are not effective against PCP [11]. The first-line treatment for PCP is the combination of the anti-folate inhibitors trimethoprim-sulfamethoxazole (TMP-SMX) together with corticosteroids to reduce destructive inflammation. There are significant problems associated with this regimen such as treatment failures and severe rash, fever, and neutropenia that often necessitate a change to alternative treatment. Treatment with corticosteroids is a double-edged sword. While it is recommended for hypoxemic patients with PCP [16], chronic steroid usage is associated with a higher rate of colonization and with mortality, especially in non-HIV patients [14]. Second-line therapies such as clindamycin-primaquine, atovaquone, or pentamidine have high rates of relapse and recurrence. Pentamidine has significant side effects, including nephrotoxicity. Currently, there are no new drugs for PCP in the pipeline. Recent studies of the new class of antifungals, the echinocandins, have shown selective elimination of the cyst form, with large populations of trophic forms remaining after treatment [9]. Although clinical use of these compounds has met with mixed results, it is possible that a combination of an echinocandin with lower doses of the more toxic agents to eliminate the trophic forms may provide an effective treatment.

## Do They Always Cause Disease?

All of the current information on the life cycle of *Pneumocystis* spp. has been derived from the study of organisms in the lungs of mammals with debilitated immune systems. No information is available on the life cycle in the non-immunosuppressed host, where their widespread presence has been confirmed in commercial animal colonies [17] and in the general population of human beings [13]. Current evidence suggests *Pneumocystis* can exist with little consequence to hosts with intact immune systems [13], [17], which may represent a commensal-type of relationship, although the length of resident time within the lung is not known. In commercial colonies of immune intact rats or mice, *Pneumocystis* is virtually undetectable by symptomatic manifestations. A survey of 137 rats from three different commercial vendors showed a 98% prevalence in normal healthy rats [17]. In humans, detection of *P. jirovecii* has been associated with underlying immune debilitation, but rarely in healthy populations [18].

However, colonization, as defined by the presence of *P. jirovecii* detected by PCR amplification or histological staining of resected lung tissue, oropharyngeal or nasopharyngeal washes, or bronchoalveolar fluids, can be associated with poorer outcomes for the colonized individuals. The changes in immune function that support colonization can be subtle, including pregnancy, chronic lung disease, or even immature immune systems, such as that in infants [13]. In persons suffering from chronic obstructive pulmonary disorder (COPD), *P. jirovecii* was detected by PCR amplification in resected lung tissue of 36.7% of patients with severe disease versus 9.1% of control subjects [19]. Although not the cause of mortality, colonized individuals had more severe airway obstruction than non-colonized individuals. Once thought to be a potential cause of sudden infant death syndrome (SIDS) in infants, subsequent studies of post-mortem tissue from infants who died from SIDS compared with those who died from other causes did not support these earlier findings, although approximately one-third of each group had detectable *P. jirovecii* [20].

It is likely that the organism replicates at low levels within an immune intact host, evading the immune system by its vast repertoire of surface antigens [13]. The balance between the mammalian host and resident or transient *Pneumocystis* populations may occur indefinitely until it is perturbed by debilitation of the immune system, induced by various means including infectious or immunosuppressive agents, congenital defects, or malnutrition, which can then lead to proliferation within the lung alveoli and eventually a lethal pneumonia if untreated. These fungi could be considered both opportunistic in self-limiting infections and pathogenic, taking advantage of the loss of the host’s immune system to increase in number, which ultimately leads to the host’s demise. However, it appears that host death is not the intent, since *Pneumocystis* rely on the host for proliferation and transmission and probably have evolved to cause as little damage as possible to this relationship to ensure their survival.

## What Is the Answer to the Question Posed in the Title?

I would argue the answer is (d). The species specificity and host-obligate nature of the members of this genus argue for a co-evolution with their respective mammalian hosts in which their preferred life style seems to be commensal like. In immunologically intact mammals, *Pneumocystis* spp. exert little to no pathogenic effects and enjoy widespread distribution among their host of choice. Since the mammalian host appears to be necessary for *Pneumocystis* spp. survival and complete life cycle including sexual replication, it would seem advantageous to keep it alive. Should the host lose some immune function, by disease or chemotherapeutic agents, the organisms can take advantage of the decreased host defenses, as would an opportunist, and enter into a more aggressive state with detectable colonization, which in some cases can be associated with clinical symptoms. This phase can be self-limiting if the immune function does not further decline, or if the source of immunosuppression is alleviated. More sustained or severe loss of immune competence by the host permits the organisms to expand and more extensively invade the lung, resulting in pneumonia and associated pathogenesis. *Pneumocystis* is opportunistic in the sense that it takes advantage of the change in the host immune response to grow and expand, but the apparent lack of clinical consequences in immunologically intact hosts, the host specificity, and the lack of innate virulence factors suggest that these fungi have adapted to form a compatible relationship rather than a pathogenic one within their natural habitat, the immune intact mammalian host.
